# Uptake of Cervical Cancer Screening and Its Barriers Using Health Belief Model Among Health Professionals Working in Public Hospitals in South Gondar Zone, Northcentral Ethiopia: Multicenter Cross-Sectional Study

**DOI:** 10.1089/whr.2023.0030

**Published:** 2024-02-22

**Authors:** Tigabu Munye Aytenew, Yohannes Tesfahun Kassie, Solomon Demis Kebede

**Affiliations:** ^1^ Department of Nursing, College of Health Sciences, Debre Tabor University, Debre Tabor, Ethiopia.; ^2^ Department of Pediatrics and Neonatal Nursing, College of Health Sciences, Debre Tabor University, Debre Tabor, Ethiopia.

**Keywords:** cervical cancer, cervical cancer screening, health professionals, public hospitals

## Abstract

**Background:**

Cervical cancer is a malignant neoplasm that originates in the cervix, and it is a leading cause of mortality, with 270,000 deaths every year globally. Of these, 85% occur in developing countries, including Ethiopia. Routine cervical cancer screening and early treatment can prevent up to 80% of cervical cancers. Health professionals are expected to screen for and be screened for cervical cancer. However, there is limited information about the uptake of cervical cancer screening among health professionals in the study area.

**Objective:**

This study aimed to determine the magnitude of cervical cancer screening uptake and identify its barriers among health professionals.

**Methods:**

A multicenter cross-sectional study design was conducted among health professionals from December 01 to 30, 2022. A total of 164 respondents were included in the study, and simple random sampling was used to select the respondents. Variables with a *p*-value of <0.05 at 95% confidence interval (CI) were considered significantly associated with the outcome variable.

**Results:**

Of the total respondents, 112 (68.3%) were younger than the age of 30 years, with a mean age of 29.4 years ranging from 21 to 45 years. Seventy-nine of the respondents (48.2%) have work experience of 6–10 years, and 103 (62.8%) are nurses in profession. In this study, the magnitude of cervical cancer screening uptake was 28.1% (95% CI: 27.7%–35.6%). Moreover, attitude (adjusted odds ratio [AOR] = 3.3, 95% CI: 2.1–5.1), age at first sexual intercourse (AOR = 2.1, 95% CI: 1.3–3.4), having history of sexually transmitted infections (STIs; AOR = 3.6, 95% CI: 1.5–11.6), knowing someone who had been screened (AOR = 2.9, 95% CI: 1.8–4.8), and cervical cancer screening training (AOR = 1.6, 95% CI: 1.1–2.9) were significantly associated with cervical cancer screening.

**Conclusion:**

Generally, this study reported that the magnitude of cervical cancer screening uptake was low. The study also indicated that attitude, age at first sexual intercourse, history of STIs, knowing someone who had been screened, and training of cervical cancer screening were independent predictors of uptake of cervical cancer screening.

## Background

Cervical cancer is the fourth-most frequent cancer in women, with an estimated 570,000 new cases representing 6.6% of all female cancers globally.^1^ It is also a leading cause of mortality, with 270,000 deaths every year; of these, 85% are in developing countries.^2^ It is a relatively rare disease in countries that have national screening programs and quality control with appropriate monitoring and evaluation.^3^ In the United States, deaths from cervical cancer reduced by 50% between 1975 and 2016, due to earlier detection of the cervical cancer.^4^

Each year, 348 new cases of cervical cancer are diagnosed in sub-Saharan Africa, and 225 women die from the disease per 1,000,000 women, accounting for 22% of all global cervical cancer.^5^ In Ethiopia, 7000 new cases and 4884 deaths of cervical cancer occur annually.^6,7^ An annual report compiled by Tikur Anbessa specialized referral hospital showed that cervical cancer accounts for around 30.3% of all cancer cases diagnosed in the hospital.^7^

Routine cervical cancer screening, and early treatment can prevent up to 80% of cervical cancers if abnormalities are identified at stages when they can be easily treated.^8,9^ PAP smear test is a screening test that checks the presence of cancer or precancerous cells in the cervix.^10^ More than 80% of cancers in sub-Saharan Africa are detected at a late stage, which is associated with low survival rates after surgery or radiotherapy; it is also associated with lack of and/or limited treatment modalities, too expensive and inaccessible for many women in low-resource countries, including Ethiopia.^11^

Studies revealed that women with a diagnosis of cervical cancer experienced physical, psychosocial, financial, and emotional burdens. This is due to surgical morbidity and chemotherapy toxicity, loss of fertility, changes in body image, sexual concerns, and altered relationships.^12,13^ Radiotherapy as part of treatment has the highest risk of long-term dysfunction of the bladder, bowel, sexual dysfunction, and psychosocial consequences.^14^ In terms of economy, the study shows that the mean outpatient cost per patient for cervical cancer is $407.2.^1,15^

Investigating the uptake of cervical cancer screening among health professionals is very important to show the gap in screening and to identify its barriers before investigating it among the community at large. Unless we investigate it among health professionals first and apply all the necessary measures based on the possible findings, it is difficult to say that health professionals have a good level of knowledge, positive attitude, and increased uptake of cervical cancer screening for themselves, which can also help them to increase the knowledge, change the attitude, and enhance the uptake of cervical cancer screening for the community at large to prevent and treat it early when it occurs.

However, there was limited information about the uptake of cervical cancer screening and its barriers among health professionals in the area, and the country at large. Therefore, this study aimed to assess the uptake of cervical cancer screening and its barriers among health professionals working in public hospitals in South Gondar Zone, Northcentral Ethiopia, 2022.

## Objective

To determine the magnitude of cervical cancer screening uptake and identify its barriers among female health professionals working in public hospitals, South Gondar Zone, Northcentral Ethiopia, 2022.

## Methods

### Study design, area, and period

A multicenter cross-sectional study design was conducted among female health professionals working in public hospitals, South Gondar Zone from December 01 to 30, 2022.

### Source and study population

All female health professionals working in all public hospitals in South Gondar Zone were the source population, whereas all female health professionals working in the three selected public hospitals were the study population.

### Inclusion and exclusion criteria

Female health professionals whose age is ≥20 years were included in the study, whereas those who had a history of cervical cancer, hysterectomy, and who had no history of sexual intercourse were excluded from the study.

### Sample size determination and sampling procedure/technique

The sample size (*n*) was calculated by computer-based Epi Info 7 software using a single-population proportion at 95% confidence interval (CI), with a 5% margin of error, and by assuming the magnitude of cervical cancer screening uptake to be 11.4%.^2^

Based on this assumption, the sample size (*n*) for the study was calculated as follows:

$$n=\frac{{\left(Z\alpha ∕2\right)}^{2}P\left(1-P\right)}{d^{2}}$$

where *n* = the minimum sample size required for the study; *Z* = standard normal distribution (*Z* = 1.96) with 95% CI; *P* = magnitude of cervical cancer screening uptake (11.4% = 0.114); and *d* = tolerable margin of error (*d* = 5% = 0.05).

$$n=\frac{{\left(1.96\right)}^{2}0.114\left(1-0.114\right)}{{\left(0.05\right)}^{2}}$$

*n* = 156. Then, by adding a 10% (0.1) nonresponse rate, the final sample size (*n*) was calculated to be 172 for this study.

Three public hospitals (Debre Tabor comprehensive specialized hospital [DTCSH], Addis Zemen primary hospital, and Mekane-Eyesus primary hospital) were selected among the eight public hospitals (one referral hospital, and seven primary hospitals) purposively for this study. Then, the final sample size was allocated for each hospital proportionally (DTCSH = 114, Addis Zemen = 30, and Mekane-Eyesus = 28). The respondents were also selected using a simple random sampling technique from each hospital.

### Dependent variable

Cervical cancer screening uptake.

### Independent variables

Sociodemographic factors (age, marital status, educational status, profession, work experience, workplace, and income), knowledge (a total of 10 questions), and attitude of the respondents (with a total of 24 Likert scale questions [strongly disagree = 1, disagree = 2, neutral = 3, agree = 4, and strongly agree = 5]). The total score of Likert scale questions was calculated, and then, the mean score was also computed to determine the level of attitude.

Sexual and reproductive health (RH) factors (early sexual intercourse, multiple sexual partners, sexually transmitted infections [STIs], and oral contraceptive pills).

Environmental factors (working facility, availability of screening service).

Information-related factors (know someone who has been screened).

### Operational definitions

#### Cervical cancer screening uptake

Those respondents who have ever been screened for cervical cancer within the past 5 years were regarded as having cervical cancer screening uptake, whereas those who have never been screened within the past 5 years were regarded as having no screening uptake.^16,17^

#### Knowledge

Respondents who had answered ≥70% (≥7/10) of the given knowledge-related questions were said to have good knowledge, whereas those who have answered <70% (≤7/10) of the given knowledge-related questions were said to have poor knowledge.^16,17^

#### Attitude

Respondents who scored ≥ the mean score (47.2) of Likert-scale questions that used to assess nurses' attitude were said to have a positive attitude, whereas those who scored < the mean score (47.2) were said to have a negative attitude toward cervical cancer screening.^16,17^

### Data collection tool and procedures

A structured and pretested self-administered questionnaire was used to collect the data. The questionnaire was adapted by reviewing different literatures,^2,6,16–19^ and it was prepared in English language. The questionnaire contains questions related to sociodemographic characteristics, sexual and RH factors, environmental factors, knowledge, and attitude. Reliability of the tool was also established with a reliability coefficient (Cronbach's alpha score) of 0.82 for knowledge-related questions and 0.86 for standardized Likert-scale questions to assess the attitude of respondents. Before data collection, training was given for both the data collectors and supervisors. Before giving the questionnaire, the data collectors have informed the respondents about the aims/purposes, risks, and possible benefits of the study, the rights and refusals to participate in the study, and the collected information would be kept confidential.

Those who were willing and had signed the informed voluntary consent form were requested to fill out the questionnaire. The data collection was held from December 01 to 30, 2022.

### Data quality control, processing, and analysis

Five percent of the questionnaires were pretested in Koladiba primary hospital to assess the reliability, clarity, sequence, consistency, understandability, and the total time that it could take to finish the questionnaire before the actual data collection. Then, the necessary comments and feedback were incorporated in the final tool to improve its quality. Two trained degree nurses were involved in the coordination of the data collection process.

Training was given for both data collectors and supervisors regarding the objective of the study, data collection tools, ways of data collection, checking the completeness of the data collection tool, and how to maintain confidentiality. Proper coding and categorization of data were maintained for the quality of the data to be analyzed.

The collected data were checked for completeness, accuracy, cleaned and coded manually, and then entered into Epi-Data version 4.2. A double data entry was done to check its validity and compare it with the original data, and then exported to Stata version 14 for analysis. Outliers had also been checked, and simple frequencies and cross-tabulations were done for missing values and variables.

A descriptive analysis was conducted to summarize the data, and the final result was interpreted in the form of text, figure, and tables. Binary logistic regression was used to identify the barriers to cervical cancer screening uptake. Bivariate and multivariable analyses were done to see the association between the outcome variable and each independent variable. The assumptions of binary logistic regression were checked, and the goodness of fit was tested by Hosmer–Lemeshow statistic and Omnibus tests.

All variables with a *p*-value of <0.2 in the bivariate analysis were entered into the final multivariable analysis model to control all possible confounders, and the variables were selected by the enter method. An adjusted odds ratio (AOR) along with 95% CI was estimated to identify the barriers to cervical cancer screening uptake among the respondents using multivariable analysis. Variables with a *p*-value of <0.05 were considered significantly associated with the outcome variable.

### Ethical consideration

Ethical clearance was obtained from Debre Tabor University, College of Health Sciences, Ethics Review Board. All the respondents were informed about the purpose of the study, their right to refuse, and written and signed voluntary consent was obtained from all respondents before data collection. The participants were told that the information obtained from them would be treated with complete confidentiality and would not cause any harm.

## Results

Of the total of 172 respondents, 164 were included in the final analysis, giving a response rate of 95.4%.

### Sociodemographic characteristics

Of the total respondents, 112 (68.3%) were younger than the age of 30 years, with the mean age of 29.4 years ranging from 21 to 45 years. Moreover, 79 (48.2%) of the respondents have work experience of 6–10 years; and about two-third, 103 (62.8%), are nurses in profession (Table 1).

**Table 1.** Sociodemographic Characteristics of the Respondents Working in Public Hospitals in South Gondar Zone, Northcentral Ethiopia, 2022 (*n* = 164)

Variables	Category	Frequency	Percentage (%)
Age	<30	112	68.3
	30–35	27	16.5
	˃35	25	15.2
Work experience	1–5 Years	58	35.4
	6–10 Years	79	48.2
	>10 Years	27	16.4
Profession	Nurse	103	62.8
	Midwife	22	13.4
	Others	39	23.8
Educational status	Diploma	67	40.9
	BSc	91	55.5
	MSc	6	3.6
Husband's educational status	Diploma and below	9	5.5
	Degree and above	155	94.5
Husband's job	Government employee	123	75.0
	Private	41	25.0

Others, physicians, laboratory technicians, anesthetists, dentists, and radiographers.

### Magnitude of cervical cancer screening uptake

In this study, the magnitude of cervical cancer screening uptake among health professionals was 46 (28.1%; 95% CI: 27.7%–35.6%) (Table 2).

**Table 2.** Magnitude of Cervical Cancer Screening Uptake Among Health Professionals Working in Public Hospitals in South Gondar Zone, Northcentral Ethiopia, 2022 (*n* = 164)

Variables	Category	Distribution of screening uptake		Cervical cancer screening uptake	
		No (%)	Yes (%)	No (%)	Yes (%)
Age	<30	85 (75.9)	27 (24.1)	85 (72.0)	27 (58.7)
	30–35	19 (70.4)	8 (29.6)	19 (16.1)	8 (17.4)
	≥35	14 (56.0)	11 (44.0)	14 (11.9)	11 (23.9)
Work experience	1–5	51 (87.9)	7 (12.1)	51 (43.2)	7 (15.2)
	6–10	57 (72.2)	22 (27.8)	57 (48.3)	22 (47.8)
	≥10	10 (37.0)	17 (63.0)	10 (8.5)	17 (37.0)
Profession	Nurse	69 (67.0)	34 (33.0)	69 (58.5)	34 (73.9)
	Midwife	17 (77.3)	5 (22.7)	17 (14.4)	5 (10.9)
	Others	32 (82.1)	7 (17.9)	32 (27.1)	7 (15.2)
Knowledge	Poor knowledge	65 (85.5)	11 (14.5)	65 (55.1)	11 (23.9)
	Good knowledge	53 (60.2)	35 (39.8)	53 (44.9)	35 (76.1)
Attitude	Negative attitude	61 (78.2)	17 (21.8)	61 (51.7)	17 (37.0)
	Positive attitude	57 (66.3)	29 (33.7)	57 (48.3)	29 (63.0)
Age at first sexual intercourse	<20	36 (52.9)	32 (47.1)	36 (30.5)	32 (69.6)
	≥20	82 (85.4)	14 (14.6)	82 (69.5)	14 (30.4)
Use of oral contraceptives	Yes	48 (65.8)	25 (34.2)	48 (40.7)	25 (54.3)
	No	70 (76.9)	21 (23.1)	70 (59.3)	21 (45.7)
History of STI	Yes	3 (50.0)	3 (50.0)	3 (2.5)	3 (6.5)
	No	115 (72.8)	43 (27.2)	115 (97.5)	43 (93.5)
Knowing someone screened	Yes	30 (57.7)	22 (42.3)	30 (25.4)	22 (47.8)
	No	88 (78.6)	24 (21.4)	88 (74.6)	24 (52.2)
Cervical cancer screening training	Yes	9 (60.0)	6 (40.0)	9 (7.6)	6 (13.0)
	No	109 (73.2)	40 (26.8)	109 (92.4)	40 (87.0)

Others, physicians, laboratory technicians, anesthetists, dentists, and radiographers.

STI, sexually transmitted infection.

Of those who had been screened, 35 (76.1%) had been screened once, and 43 (93.5%) and 3 (6.5%) had negative and positive results, respectively. In addition, among those who had been screened, the majority, 29 (63.0%), of them did not know the methods of cervical cancer screening.

### Barriers of cervical cancer screening uptake

Of the total respondents, 88 (53.7%) had a good level of knowledge. Similarly, 85 (51.8%) also had a positive attitude toward cervical cancer screening practice. On the contrary, the majority of respondents, 149 (90.9%), did not get cervical cancer screening training (Table 3).

**Table 3.** Barriers of Cervical Cancer Screening Uptake Among Health Professionals Working in Public Hospitals in South Gondar Zone, Northcentral Ethiopia, 2022 (*n* = 164)

Variables	Category	Frequency	Percentage (%)
Knowledge	Poor knowledge	76	46.3
	Good knowledge	88	53.7
Attitude	Negative attitude	79	48.2
	Positive attitude	85	51.8
Age at first sexual intercourse	<20	68	41.5
	≥20	96	58.5
Use of oral contraceptives	Yes	73	44.5
	No	91	55.5
History of STI	Yes	6	3.7
	No	158	96.3
Knowing someone screened	Yes	52	31.7
	No	112	68.3
Cervical cancer screening training	Yes	15	9.1
	No	149	90.9

### Reasons for not being screened for cervical cancer

**FIG. 1.**
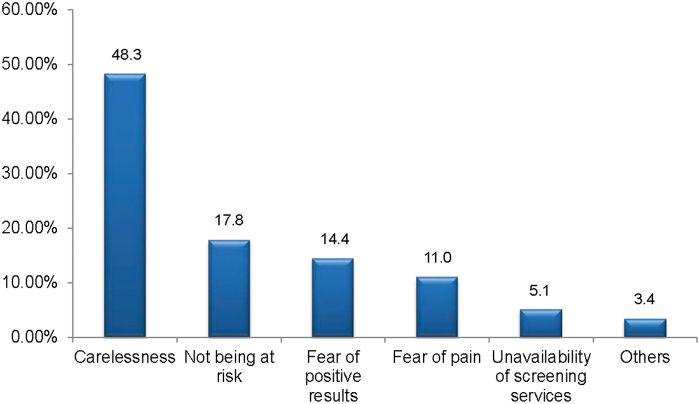
Reasons for not being screened for cervical cancer among health professionals working in public hospitals in South Gondar Zone, Northcentral Ethiopia, 2022 (*n* = 164).

The most common reasons for not being screened were carelessness, 57 (48.3%), and perceiving that they are not at risk, 21 (17.8%) (Fig. 1).

### The association between cervical cancer screening uptake and independent variables

A total of nine variables (work experience, profession, knowledge, attitude, age at first sexual intercourse, use of oral contraceptives, history of STI, knowing someone who had been screened, and cervical cancer screening training) were included in the final multivariable analysis. In the multivariable analysis, respondents with a positive attitude toward cervical cancer screening uptake were 3.3 times more likely to be screened than those with a negative attitude (AOR = 3.3, 95% CI: 2.1–5.1).

Likewise, respondents who had first sexual intercourse at the age of <20 years were also 2.1 times more likely to be screened for cervical cancer compared with respondents who had sexual intercourse at the age of 20 years or older (AOR = 2.1, 95% CI: 1.3–3.4). Similarly, respondents with a history of STI were 3.6 times more likely to be screened than those who did not have a history of STI (AOR = 3.6, 95% CI: 1.5–11.6).

Moreover, those respondents who knew someone who had screened for cervical cancer were also 2.9 times more likely to be screened for cervical cancer than those who did not know (AOR = 2.9, 95% CI: 1.8–4.8). Likewise, those respondents who had received cervical cancer screening training were 1.6 times more likely to be screened for cervical cancer compared with respondents who had not received cervical cancer screening training (AOR = 1.6, 95% CI: 1.1–2.9) (Table 4).

**Table 4.** Showing the Association Between Independent Variables with Cervical Cancer Screening Uptake Among Health Professionals Working in Public Hospitals in South Gondar Zone, Northcentral Ethiopia, 2022 (*n* = 164)

Variables	Category	Uptake		OR (95% CI)		*p*
		No	Yes	COR	AOR	
Age	<30	85	27	1	1	
	30–35	19	8	1.1 (0.7–1.9)	0.9 (0.5–1.8)	
	≥35	14	11	2.7 (1.6–4.4)	1.7 (0.8–3.4)	
Work experience	1–5	51	7	1	1	
	6–10	57	22	1.4 (0.9–2.2)	1.2 (0.7–2.0)	
	≥10	10	17	2.6 (1.5–4.5)	1.5 (0.7–3.4)	
Profession	Nurse	69	34	0.6 (0.4–1.7)	0.6 (0.3–1.6)	
	Midwife	17	5	0.7 (0.4–1.3)	0.7 (0.3–1.4)	
	Others	32	7	1	1	
Level of knowledge	Poor knowledge	65	11	1	1	
	Good knowledge	53	35	0.5 (0.3–1.2)	0.5 (0.3–1.1)	
Level of attitude	Negative attitude	61	17	1	1	
	Positive attitude	57	29	3.5 (2.4–5.3)	3.3 (2.1–5.1)	0.001
Age at first sexual intercourse	<20	36	32	1.6 (1.1–2.3)	2.1 (1.3–3.4)	0.002
	≥20	82	14	1	1	
Use of oral contraceptives	Yes	48	25	1.7 (0.9–1.4)	1.6 (0.7–1.3)	
	No	70	21	1	1	
History of STI	Yes	3	3	2.7 (1.1–6.8)	3.6 (1.5–11.6)	0.007
	No	115	43	1	1	
Knowing someone screened	Yes	30	22	4.3 (2.9–6.3)	2.9 (1.8–4.8)	0.001
	No	88	24	1	1	
Cervical cancer screening training	Yes	9	6	1.5 (1.2–3.2)	1.6 (1.1–2.9)	0.001
	No	109	40	1	1	

AOR, adjusted odds ratio; CI, confidence interval; COR, crude odd ratio.

## Discussion

Investigating the uptake of cervical cancer screening among health professionals is very important to show the gap of screening service utilization and its barriers before investigating it among the community at large. Unless we investigate it among professionals and implement all the necessary measures based on the possible findings to scale-up the uptake of cervical cancer screening by intervening at the barriers, such as changing their level of knowledge and attitude and providing cervical cancer screening training, we will not be able to scale-up the uptake of cervical cancer screening.

This study revealed that the magnitude of cervical cancer screening uptake among health professionals was 28.1%. The study also found that attitude toward cervical cancer screening uptake, age at first sexual intercourse, history of STI, knowing someone who had been screened for cervical cancer, and training of cervical cancer screening were significantly associated with the uptake of cervical cancer screening.

In this study, the magnitude of cervical cancer screening uptake among health professionals was 28.1%. This finding is lower than the Federal Ministry of Health-Ethiopia, National cervical cancer prevention strategic plan (80%).^20^ However, it was higher than the studies conducted among health professionals in Saudi Arabia (20.6%),^21^ Uganda (19%),^22^ Mekele city (10.7%),^16^ Southern Ethiopia (11.4%),^2^ and Addis Ababa (17%).^23^ The reason for the difference in cervical cancer rates could be because of the varying study periods and the amount of information provided through different media channels. The government is taking notice of this and is creating national policies and strategies to prevent and control cervical cancer.

This study showed that those respondents who had a positive attitude were 3.26 times more likely to be screened compared with those who had a negative attitude toward cervical cancer screening. This finding is similar to studies conducted in Nepal,^18^ Mekele,^16^ and Debre Markos.^23^ This might be due to the fact that having a positive attitude toward cervical cancer screening may trigger or initiate for cervical cancer screening uptake.

This study also indicated that respondents who had first sexual intercourse at the age of <20 years were 2.1 times more likely to be screened for cervical cancer compared with respondents who had sexual intercourse at the age of 20 years or older. This study is in line with studies conducted in Montero,^8^ Rwanda,^24^ Japan,^25^ and India.^26^ The reason could be some people might be at a higher risk of sexually transmitted infections (STIs) due to engaging in sexual activity at an early age without being married. This can lead to having multiple sexual partners and an increased likelihood of contracting an STI. As a result, these individuals may choose to get screened for STIs.

Similarly, this study indicated that respondents with a history of STIs were 3.6 times more likely to be screened than those who did not have. This finding is supported by studies conducted in India, Turkey, and Debre Markos, Ethiopia.^18,21,27^ This might be due to the fact that respondents who had been exposed to STI and become symptomatic are more likely to receive medical care, and by being in the medical care system, their likelihood of screening would be higher. On the contrary, this study showed that those respondents who knew someone who had been screened were also 2.9 times more likely to be screened for cervical cancer than those who did not know. This report is similar to studies conducted in Uganda^9^ and Debre Markos.^18^ This might be due to information sharing, which might have, in turn, triggered them to be screened.

Moreover, this study reported that those respondents who received cervical cancer screening training were 40% more likely to be screened for cervical cancer; this might be due to the fact that the training could have changed the attitude, knowledge toward cervical cancer screening, and the magnitude of uptake among the health professionals easily.

### Limitations of the study

Since the design was cross-sectional, it cannot be used to analyze the uptake over a period of time. In addition, the respondents might also be subjected to recall and social desirability biases.

## Conclusion

Generally, the findings of this study reported that the magnitude of cervical cancer screening uptake among female health professionals was low. The study also indicated that attitude toward cervical cancer screening, age at first sexual intercourse, history of STI, knowing someone who had been screened for cervical cancer, and cervical cancer screening training were independent predictors of cervical cancer screening uptake among female health professionals working in South Gondar Zone public hospitals.

### Recommendations

(1) To detect and manage cervical cancer early, the Ministry of Health, Amhara Regional Health Bureau, Zonal Health Department, nongovernmental organizations (NGOs), and other responsible stakeholders must increase their emphasis on cervical cancer screening uptake among female health professionals, and the community at large.
(2) Those female health professionals shall improve their knowledge, attitude, and uptake of cervical cancer screening to detect and manage cervical cancer at the early stage.
(3) Cervical cancer screening experts and mass media shall strengthen their roles to make health professionals, and the community at large, to be well informed about cervical cancer and its screening.
(4) Other researchers should conduct further studies using different tools and study designs to show the gaps clearly and identify additional factors.

## Consent for Publication

Not applicable.

## Data Availability

All the data used for the study were included in the article.

## Abbreviations Used

AOR: adjusted odds ratio
CI: confidence interval
DTCSH: Debre Tabor comprehensive specialized hospital
RH: reproductive health
STI: sexually transmitted infection
